# The Spherical Tokamak for Energy Production: theme issue introduction

**DOI:** 10.1098/rsta.2023.0416

**Published:** 2024-08-26

**Authors:** I. T. Chapman, S. C. Cowley, H. R. Wilson

**Affiliations:** ^1^ UK Atomic Energy Authority, Culham Campus, Abingdon, Oxfordshire OX14 3DB, UK; ^2^ Princeton Plasma Physics Laboratory, 100 Stellarator Road, Princeton, NJ 08540, USA; ^3^ Oak Ridge National Laboratory, Oak Ridge, TN 37831, USA

**Keywords:** fusion energy, magnetic confinement fusion, plasma physics

## Abstract

This theme issue collects together papers summarising the conceptual design of the Spherical Tokamak for Energy Production (STEP). In 2019, the UK government funded the first design stages of a prototype fusion powerplant based on a compact toroidal geometry, called STEP. The primary technical aims of STEP are to produce net energy, to be self-sufficient in tritium fuel and to demonstrate a maintenance regime that would extrapolate to appropriate availability for commercial powerplants. After 5 years and over 1000 person-years of detailed scientific and engineering conceptual design, this theme issue acts as a compendium of the current design basis for STEP, noting that this is a snapshot in time and that the design will continue to evolve.

This article is part of the theme issue ‘Delivering Fusion Energy – The Spherical Tokamak for Energy Production (STEP)’.

## 1. The case for fusion energy

Analysis of global progress to reach net-zero greenhouse gas emissions shows that current decarbonization plans will not achieve the aim by 2050 [1] and that real implementation lags even further behind. Although the proportion of energy generated from low-carbon sources is increasing, so is the absolute quantity of greenhouse gases entering the atmosphere as global energy use increases [1]. Limiting global energy demand is probably unrealistic and, importantly, inequitable. Population growth in the region of 20% by approximately 2100 [2] will drive demand and the average consumption of energy by those in emerging economies should reasonably be expected to rise to enable growth in living standards. The realistic deployment of current low-carbon sources is more constrained than often appreciated [3] owing to land use, environmental unsuitability and the need for related infrastructure (grid and storage). Indeed, while existing sources of zero-carbon electricity can reduce carbon emissions efficiently, reaching net-zero emissions with existing technology is predicted to be exorbitantly expensive (refer e.g. [4], where full decarbonization could yield costs exceeding $220/MWh). Therefore, there is a strong case for seeking additional new, broadly deployable ‘firm’ (available on demand) energy sources. Further, many industrial processes are not amenable to efficient decarbonization through electricity as they need high-grade (temperature) heat.

Fusion energy has the potential to fill the gap in the availability of firm energy sources and to make net-zero attainable [5]. It could be an important option in a sustainable energy portfolio to replace fossil fuels as the world’s primary energy source. Fusion would provide predictable power generation with no carbon emissions, fuel sources lasting for millions of years and many natural safety features. Fusion requires low land use, has a high energy yield, and suitably designed power plants would produce little very long-lived radioactive waste. In short, it is a highly attractive energy source. However, fusion is notoriously difficult to achieve in a controlled, continuous fashion on Earth. Fusion occurs when reactant ions overcome the strong repulsive coulomb force between them and come within a nuclear separation of one another, binding via the strong nuclear force to form new products. For elements lighter than iron, there is a deficit in mass across the reaction which is converted into kinetic energy carried in the reaction products. It is this energy which can be harnessed for electricity and/or heat production. The prerequisite to overcome the coulomb barrier necessitates sufficient kinetic energies of the reactants, and for a high enough reaction rate this requires high temperatures (of the order 100 x 10^6^ K). The attractive properties of fusion combined with the imperative to address climate change has resulted in a burgeoning interest in the field with a notable recent growth in privately funded fusion ventures seeking to accelerate its delivery.

Delivering a viable fusion power plant is one of the holy grails of science and engineering but it represents an unprecedented technical challenge. These engineering challenges are numerous, and must be met simultaneously: (i) the sustainment of a controlled plasma over long timescales with fusion-born helium dominating the plasma heating; (ii) the controlled exhaust of heat and helium ‘ash’ from the plasma core; (iii) the development of structural materials which have to sustain, for many years, large forces and pressures in the presence of exceptionally intense neutron fluxes, while minimizing volumes of radioactive waste; (iv) the ability to generate its own tritium fuel; and (v) the requisite high availability and efficiency of the machine and its systems to produce a viable levelized cost of electricity. Fusion is different from most other technologies in that the validation of components in a representative environment is only possible in a complete fusion device and probably a complete plant. Furthermore, the cost and timescale of each step means that working towards the final commercial product via a succession of small-increment full physical prototypes is unacceptable, given the urgency of addressing climate change. Making large steps leads to two additional challenges: (vi) development of extensive reliable physics-based models and an advanced computing programme for optimization towards robust, low-uncertainty predictions of the plasma and materials performance; and (vii) comprehensive *in silico* design, digital prototypes (‘tested’ in virtual environments), and finally models of components and sub-systems to support a convincing qualification case.

The extraordinary conditions required for fusion have been achieved since the mid-1990s. Numerous landmark results in the last 2 years have further boosted optimism. In 2024, it was announced that the JET device achieved a record 69 MJ of fusion energy [6]. While the fusion reaction was only sustained for 5 s owing to the limitation of the copper magnet coils used in JET, the experiments matched predictions made in advance which gives confidence in our designs for next-step fusion devices. In addition, researchers in China demonstrated the ability to sustain a plasma under fusion conditions for 1000 s using super-conducting magnets [7]. In a complementary approach to fusion energy, the Lawrence Livermore National Laboratory recently demonstrated fusion ignition [8], whereby more energy from fusion was released than the laser energy absorbed by the fuel to drive the fusion reaction. A very high-field magnet using high-temperature superconductors at a large scale has been demonstrated [9], offering hope for much-improved fusion performance in smaller machines. Finally, and most pertinently to the Spherical Tokamak for Energy Production (STEP) programme, MAST Upgrade [10] has demonstrated an order-of-magnitude reduction in the peak heat flux reaching the machine walls in a compact design [11]. Notwithstanding these advances, each discrete challenge in fusion is significant and solving these concurrently, in a single design that produces net energy at a meaningful level of plant availability, and that is able to generate sufficient tritium fuel (all essential characteristics of eventual power plants) remains unsolved and extremely difficult. For this reason, it is important to state that fusion is very unlikely to make a contribution to reducing carbon dioxide production much before 2050. However, increasing energy demand beyond 2050 as well as the inefficiency of replacing high-grade heat sources with present low-carbon alternatives means new solutions are required to plug the widening supply–demand gap in addition to the maximum deployment of current renewables and civil nuclear power.

## 2. The global fusion landscape

The largest current fusion programme is the delivery of ITER [12,13], a global collaboration of 35 countries aiming to demonstrate 500 MW of fusion power released while using 50 MW of injected heating power to achieve fusion conditions. Although ITER is not designed to produce net energy it is the world’s first industrial-scale fusion programme and has already driven substantial development of supply chains globally. This, together with the landmark results noted above, and the increase in market appetite for investment in fusion, has resulted in the publication of a number of government strategies in recent years. The most established public programmes are the coordinated European effort [14] to design a demonstration power plant, DEMO [15–17]; the UK fusion strategy [18] including a unique approach to a public–private partnership [19]; the US National Academies report [20], which stimulated a White House ‘bold decadal vision’ for fusion [21] and the subsequent public–private partnership programme [22]; and the Chinese Fusion Engineering Test Reactor [23] now with a broad range of participants under state control [24]. All of these programmes aim for prototype power plants to commence operations before the middle of the century. Beyond these well-established strategies, other countries have indicated increased support for fusion development, especially through public–private partnerships, such as in Germany [25] and Japan [26].

While it is probable that state support will be needed for the delivery of the first prototypes and demonstration plants, there is a growing number of private fusion companies. Indeed, over $6 billion has now been invested in more than 40 companies worldwide, with 93% of these private fusion companies aiming to deliver a fusion prototype device during the 2030s [27]. The first private fusion power purchase agreement has even been made [28]; however, it is worth noting that as of today, the only fusion power plant endeavour in the world that has agreed on a site for construction is STEP, at a former coal power station in Nottinghamshire in the United Kingdom [29], which indicates the embryonic stage of power plant delivery globally.

## 3. The economics of fusion power plants

The energy produced by a fusion plant must be affordable, even if there may be national strategic drivers. The abundance and low cost required to source fuel for fusion reactors (principally deuterium and lithium to breed tritium) mean that the levelized cost of electricity (LCOE) may well be dominated by the construction cost of the plant [30] (similar to fission power plants), although this depends on the operational costs which vary between concepts. Naturally, the economic performance of future fusion plants is uncertain given the inherent technological and scientific challenges, and estimates of the LCOE for fusion plants vary. General expressions for the LCOE of a fusion plant have been provided by, for example, the PROCESS code [31–33] which shows that dominant contributions are in overnight capital costs with significant contributions in operations and maintenance, and replacement costs [31]. Principal mechanisms that are expected to influence capital cost are the size of the plant and the modularity of its fusion core. Larger plants and/or more plant cores may produce more energy but at increased capital cost. Optimized designs which use specific magnetic geometries [34,35] aim primarily to drive down the size but retain the energy yield, resulting in a lower build cost/power ratio. However, it is probable that there will be a minimum unit size in fusion for commercial viability [36,37] owing to the large recirculating power requirements of all fusion devices and the huge challenge of handling the power fluxes in compact designs. Major contributors to the operational cost are availability, cost of consumable components and the overall conversion efficiency of the plant. All of these aspects will be addressed for STEP in this theme issue.

The penetration of fusion into the market is challenging to predict when looking beyond the 2040 timescale, but one could extrapolate from other comparable large technologies, such as early adoption of oil and gas, or early adoption of conventional nuclear fission power plants. If it were valid to use such previous growth rates as a guide, fusion could reach 10% of global energy demand by 2100— equivalent to supplying all of Europe—or as much as 40% if capital costs were 30% cheaper [38]. The STEP programme was conceived to minimize the overnight capital costs by adopting a compact geometry requiring smaller magnets and smaller shielding buildings, which together account for the majority of the costs incurred in the ITER project to date.

This theme issue covers every aspect of the STEP conceptual design: Baker, Methven and Waldon explain the overall UK fusion strategy, the objectives of STEP and the trade-offs in its concept design. Meyer, Lennholm, Nasr, Lamb, Cane, Quadling, Lord and Acres outline the different aspects of the overall power plant design, while Afify discusses the approach to fusion safety and regulation. Finally, Davis, Kempton and Lux describe the future pathways to maturing the design through computational advances, engineering methodology and cost optimization, respectively.

## Data Availability

This article has no additional data.
